# Sudden Unexpected Death Caused by Cardiac Metastasization from Histiocytic Sarcoma

**DOI:** 10.3390/ijerph182412911

**Published:** 2021-12-07

**Authors:** Alessandro Feola, Paola Ciamarra, Mariavictoria De Simone, Anna Carfora, Gelsomina Mansueto, Carlo Pietro Campobasso

**Affiliations:** 1 Department of Experimental Medicine, University of Campania “Luigi Vanvitelli”, 80138 Naples, Italy; alessandro.feola@unicampania.it (A.F.); mariavictoria.desimone@studenti.unicampania.it (M.D.S.); anna.carfora@unicampania.it (A.C.); carlopietro.campobasso@unicampania.it (C.P.C.); 2 Department of Advanced Medical and Surgical Sciences (DAMSS), University of Campania “Luigi Vanvitelli”, 80138 Naples, Italy; gelsomina.mansueto@unicampania.it; 3 Clinical Department of Laboratory Services, Public Health-Legal Medicine Unit, University of Campania Luigi Vanvitelli, Via Luciano Armanni 5, 80138 Naples, Italy

**Keywords:** haematological malignancies, lymphoma, leukaemia, histiocytic sarcoma, sudden death, autopsy, arrhythmia, myocarditis

## Abstract

Background: Haematological malignancies, such as lymphoma and leukaemia, can have a variety of clinical manifestations. The most frequent cause of death from haematological malignancies is multiple organ failure due to neoplastic organ infiltration and/or septic shock. Histiocytic sarcoma (HS) is a rare malignant nodal or extranodal tumour with histiocytic immunophenotype that originates from a lymphohematopoietic precursor. The patients with HS usually have a poor prognosis due to its aggressive clinical behaviour. Rare cases of undiagnosed sudden HS death have been described in the literature. Methods: A forensic autopsy of a 46-year-old white male who died at home suddenly and unexpectedly without warning conditions or known diseases. Gross analysis, histology and toxicology were also performed. Results: The diagnosis of HS of the ileum with secondary nodal and cardiac metastatization was made. Conclusions: A prompt diagnosis of HS in life is paramount because it can make a difference in prognostic outcomes.

## 1. Introduction

Histiocytic sarcoma (HS), also known as histiocytic lymphoma, is an exceedingly rare malignant neoplasm that originates from a lymphohematopoietic precursor [1]. It is most commonly present in lymph nodes or at extranodal sites, in the gastrointestinal tract (GI), bone marrow, skin, bladder and other sites, including the head. HS is mostly located in the GI tract, with only few cases reported in the stomach [2]. Histologically, it is characterized by medium-sized neoplastic cells with an eosinophilic cytoplasm, abundant vacuoles and high mitotic rate. Characteristically, HS is aggressive, with a destructive pattern in the primitive site of onset, and while in the metastatic localization, it has an infiltrative pattern. In fact, the diagnosis of metastatic diffusion is very difficult, complex and involves a differential diagnosis with other neoplastic or not neoplastic conditions. The typical neoplastic immunophenotype is characterized by the expression of CD68, lysozyme and in addition usually leukocyte common antigen (LCA/CD45, CD45RO) [3]. It is difficult to distinguish between the primary and secondary localizations into the systemic disease. Neoplastic cells can express one or more histiocytic markers including CD68, lysozyme and CD163 and typically do not express non-Langerhans dendritic or myeloid markers despite being derived from a lymphohematopoietic precursor. Essential for the diagnosis are the positivity of the neoplastic elements for CD68 and lysozyme [3,4]. HS is generally diagnosed in life, but unexpected death can also occur in apparently healthy and totally asymptomatic individuals. A case of secondary metastasis in the heart with sudden death is reported.

## 2. Materials and Methods

A 46-year-old white male died suddenly and unexpectedly at home without warning conditions or known diseases. A forensic autopsy was performed 72 h after death in accordance with the Recommendations on the Harmonization of Forensic Autopsy Rules of the Committee of Ministers of the Council of Europe (1999).

Histology and immunohistochemistry. For each organ, sampling was carried out. After fixation in 10% neutral buffered formalin and paraffin embedding, extensive sampling of the ventricles and atria was performed. The tissues along the course of the coronary arteries with the anterior and posterior descending branches and of the septum including the conduction system were also sampled. Sections for haematoxylin–eosin staining (H&E) for morphological diagnosis and serial sections of 4 microns for immunohistochemistry (IH) were performed. Human monoclonal anti-CD68 and anti-lysozyme antibodies (1:100) with the DAB Substrate-Chromogen system and Alkaline Phosphatase/RED (DAKO) were used.

Femoral blood was analysed for alcohol (ethanol) and volatiles by headspace gas chromatography coupled with a flame ionization detector (GC/HS-FID). All post-mortem specimens (blood, urine, vitreous humour, liver, brain) were screened for the presence of the main different classes of drugs (pharmaceuticals and illegal drugs) by immunological or chromatographic methods as appropriate. A systematic toxicological analysis (STA) was performed by means of an LC–MS/MS system (API 3200 triple quadrupole ABI-SCIEX) in the multiple reaction monitoring (MRM) mode.

## 3. Results

### 3.1. Autopsy Findings

**Figure 1:**
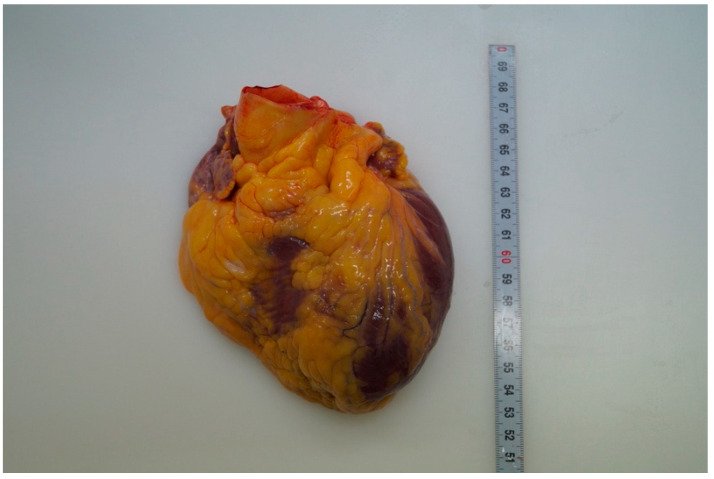
Macroscopic appearance of the heart.

No blood fluid in the abdominal or thoracic cavity, while the serous surfaces of the ileum appeared to be widely congested with blood collections in the perivisceral fat and enlarged lymph nodes. Macroscopically, the heart and other systemic organs appeared normal in diameter and features. The heart was 397 g in weight (Figure 1). After four-chamber opening, no relevant defects were observed at the examination of the valvular apparatuses, emerging vessels and cardiac cavities. The ventricular walls had normal thickness, as well as the interventricular septum. No abnormal origins of the coronary arteries were observed, along with no atherosclerotic lesions.

### 3.2. Toxicology Analysis

Toxicology analysis showed no evidence of drugs or alcohol.

### 3.3. Histology and Immunohistochemistry. Microscopic Findings

**Figure 2:**
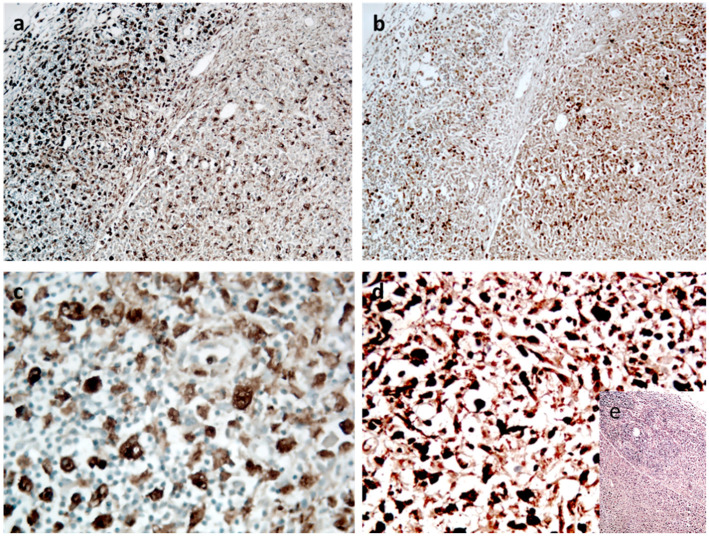
Node section: (**a**) CD68- and (**b**) lysozyme-positive cells (magnification, ×20). Details of cell cytology at ×40. In (**c**) CD68 and in (**d**) lysozyme. In (**e**), insert of H&E: subcapsular area with distorted structure and loss of germinal centres (×10 magnification) (DAB Substrate-Chromogen system).

**Figure 3:**
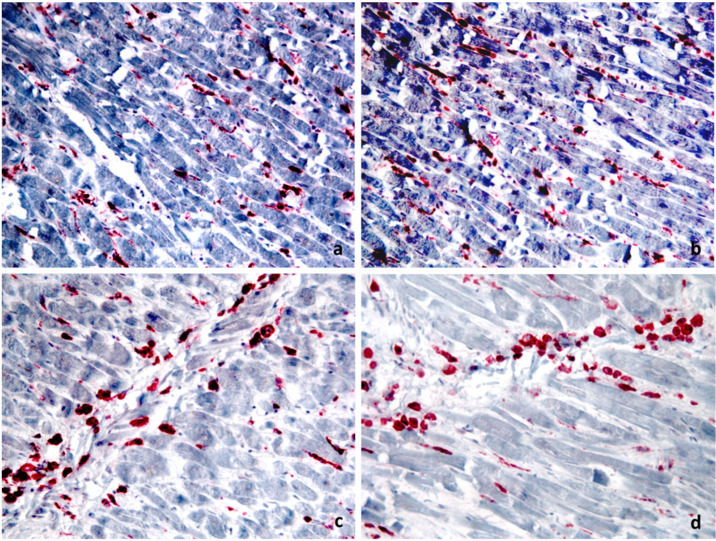
Sections from the same paraffin block of the heart demonstrate positivity for CD68 of the neoplastic elements ((**a**) ×20 magnification) and for lysozyme ((**b**) ×20 magnification). Sections from the same paraffin block but from different fields show the cytological details of the neoplastic cells for CD68 ((**c**) ×40 magnification) and for lysozyme ((**d**) ×40 magnification) (alkaline phosphatase/RED system).

The degenerative changes of the ileum mucosa with oedema, fibrosis and diffuse trans-parietal lymphoid infiltrate with associated middle perivisceral elements and absence of continuous solutions were observed. The perivisceral lymph nodes appeared subverted from predominantly atypical epithelioid or sarcomatoid medium-sized cells that were CD68- and lysozyme-positive (Figure 2), as well as CD45RO, typical markers in the differential diagnosis with other haematological diseases. The same diffuse cell population was observed in the heart (Figure 3) with prevalent interstitial and perivascular localization mimicking myocarditis. The diagnosis of HS of the ileum with secondary nodal and cardiac metastatization was made.

## 4. Discussion

Haematological malignancies, such as lymphoma and leukaemia, can have a variety of clinical manifestations. Symptoms are often caused by myeloid or lymphoid cell dysfunction or by the proliferation of neoplastic blasts in the bone marrow [5].

Among haematological malignancies, HS is classified also as part of the adult non-Hodgkin lymphomas and occurs in both sexes and in a wide age range (mean age, 46 years) [6]; neoplastic cells have the same morphology and immunohistochemical profiles as mature histiocytes, but the etiopathogenesis is undiscovered. Treatments based on chemo- and radiotherapy provide unsatisfying results. The overall survival remains short [7]; up to now, no definitive molecular alterations or treatment targets have been identified in histiocytic sarcoma.

HS is an extremely rare and aggressive disease with a rapidly progressing clinical course. In the majority of cases, the primary presentation is an extranodal mass involving other organs (spleen, liver, bone marrow, soft tissue, meninges or gastrointestinal tract, etc.) [8,9], and the most frequent findings include hepatosplenomegaly, lymphadenopathy, intestinal obstruction, rash and pancytopenia [10].

The vast majority of GI HS is located in the bowel, and few cases are found in the stomach. When the bowel is affected, the symptomatology can be made worse by perforation with bleeding and hypovolemic shock.

However, patients may be also asymptomatic or may have no warning conditions or known diseases. Rarely the diagnosis is made after death which can occur for pulmonary emboli, cardiopulmonary arrest secondary to superior vena cava syndrome, multiple organ failure, sepsis, intracranial haemorrhage or anoxic brain injury and other causes. The most frequent cause of death is multiple organ failure due to neoplastic organ infiltration and/or septic shock [4,5].

Furthermore, the secondary metastatic spread of malignant cells may result in systemic effects such as blood hypercoagulability [11,12]. Only few cases of HS with cardiac metastasisation have been described in the literature [13]. Akishima et al. [14] reported the case of a 50-year-old man with severe gastrointestinal symptoms who died due to gastrointestinal failure. HS infiltration of the spleen, liver, bone marrow and lymph nodes were reported; moreover, in this case, an infiltration to the interstitium of the epicardium, kidney and alveolar septum was detected. Haynes et al. [15] described the case of a 15-year-old female who was affected with gastrointestinal, neurologic and ocular symptoms. Many solid organs were macroscopically altered with a firm cream-white infiltrate. The heart (490 g) was grossly distorted by a firm white infiltrate throughout both atria and ventricles, and the epicardium was studded with white nodules. The infiltrate—detected throughout the full myocardial thickness—consisted of cells intervening between myocytes singly, in small clusters and in sheets with separation and destruction of the myocytes. Myocardial inflammatory infiltration may occur in association with many diseases that can be directly involved in the death process or contribute to death, and myocardial malignant cells infiltration could mimic myocarditis, [16]. In case of myocarditis, the border between physiological and pathological changes is poorly defined, and a detailed macroscopic and microscopic description of cardiac findings is necessary [16]. In fact, according to the guidelines for post-mortem investigation of sudden cardiac death recently updated by the Association for European Cardiovascular Pathology [16,17], in the absence of myocyte necrosis, small foci of inflammatory cells (even after immunohistochemistry), are not sufficient evidence of myocarditis. Only the combined detection of microfocal infiltrates in the myocardium by thorough histological and histochemical examination along with the genetic analysis of cardiotropic viruses can support the diagnosis of virus-induced myocarditis with fatal arrhythmia [18]. However, in this case, the specific morphology and immunohistochemical properties of the malignant cells characterized by atypical epithelioid or sarcomatoid CD68- and lysozyme-positive medium-sized cells are a reliable and fundamental tool for the differential diagnosis. Immunohistochemistry is a comforting method for diagnosis when morphology is not sufficient, especially in cases of myocardial damage, but also in head trauma and mechanical asphyxia [19,20,21,22]. In our case, nodal subversion together with the morphology of the visibly atypical cellular elements and therefore CD68 and lysozyme labelling (Figure 2) are consistent with a diagnosis of HS and metastatic localization of cytologically atypical elements in the myocardium.

## 5. Conclusions

In each sudden unexpected death, pathologists and physicians are requested to find evidence of a specific cause of death, and in particular to establish whether cardiac death is secondary to arrhythmic or nonarrhythmic mechanisms or otherwise to heart failure [21,22,23,24]. To the best of our knowledge, this is the first reported case of sudden death with no remarkable macroscopic findings. The patient was asymptomatic, with no other diseases. The cause of death was attributed to myocardial malignant cells infiltration mimicking myocarditis with arrythmia. Therefore, haematological malignancies should be taken into account in the differential diagnosis with viral myocarditis and sudden unexpected death.

## Data Availability

Not applicable.
